# Efficacy of mirabegron for ureteral stones: a systematic review with meta-analysis of randomized controlled trials

**DOI:** 10.3389/fphar.2023.1326600

**Published:** 2023-12-20

**Authors:** Zhenguo Wang, Junpeng Chi, Yuhua Liu, Jitao Wu, Yuanshan Cui, Chenchen Yang

**Affiliations:** ^1^ Department of Urology, The Affiliated Yantai Yuhuangding Hospital of Qingdao University, Yantai, Shandong, China; ^2^ Department of Urology, Tengzhou Central People’s Hospital, Tengzhou, China

**Keywords:** mirabegron, ureteral stones, medical expulsive therapy, effectiveness, meta-analysis

## Abstract

**Background:** Medical expulsive therapy demonstrates efficacy in managing ureteral stones in patients amenable to conservative interventions. This meta-analysis aims to evaluate the effectiveness of mirabegron in the treatment of ureteral stones.

**Methods:** From conception to November 2023, we examined PubMed databases, the Cochrane Library, Embase, Ovid, Scopus, and trial registries for this systematic review and meta-analysis. We chose relevant randomized controlled trials (RCTs) evaluating the efficacy of mirabegron as an expulsive treatment for ureteral stones. The Cochrane risk of bias method was used to assess the quality of the evidence. Outcome measures, which included the stone expulsion rate (SER), expulsion time, and pain episodes, were analyzed using RevMan 5.4 and Stata 17.

**Results:** Seven RCTs (N = 701) had enough information and were ultimately included. In patients with ureteral stones, mirabegron-treated patients had a substantially higher SER [odds ratio (OR) = 2.57, 95% confidence interval (CI) = 1.41–4.68, *p* = 0.002] than placebo-treated patients. Subgroup analysis revealed that mirabegron was superior to placebo in patients with small ureteral stones (OR = 2.26, 95% CI = 1.05–4.87, *p* = 0.04), with no heterogeneity between studies (*p* = 0.54; I^2^ = 0%). Mirabegron patients had a higher SER than the control group for distal ureteral stones (DUSs) (OR = 2.48, 95% CI = 1.31–4.68, *p* = 0.005). However, there was no difference in stone ejection time or pain episodes between groups.

**Conclusion:** Mirabegron considerably improves SER in patients with ureteral stones, and the effect appears to be more pronounced for small and DUSs. Nevertheless, mirabegron treatment was not associated with improved stone expulsion time or pain management.

## 1 Introduction

Urolithiasis is a prevalent disorder, impacting approximately one in 10 individuals globally, with an increasing incidence and prevalence. Ureteroscopy, percutaneous nephrolithotomy, extracorporeal shock wave lithotripsy (ESWL), and open and laparoscopic surgeries, along with medical expulsive therapy (MET), constitute the primary modalities for managing ureteral calculi. The advantages of MET were substantiated by a comprehensive study and meta-analysis published in 2006 (Hollingsworth et al., 2006). The primary objective of MET is to enhance the expeditious clearance of stones along the ureter, thereby preventing ureteral obstruction and alleviating ureteral colic. This approach aims to circumvent the need for surgery and more invasive interventions, both of which may entail adverse consequences.

Presently, a variety of medications are employed in MET, with the most prevalent categories including α1-adrenoceptor antagonists (commonly known as α-blockers), calcium channel inhibitors, phosphodiesterase type 5 inhibitors (PDEI-5), and non-steroidal anti-inflammatory drugs (NSAIDs). The most widely recommended drugs for MET are α-blockers, which stop the contractions of the ureteral muscle, reduce basal tone, and lessen colic discomfort and peristaltic frequency. These effects may help remove ureteral stones and may be advantageous for MET. Tamsulosin, alfuzosin, and silodosin are three forms of α-blockers used for MET, with tamsulosin being the most extensively documented in the literature (Liu et al., 2015; Huang et al., 2016). The use of the α-blocker tamsulosin is supported by current North American and European treatment recommendations, with the most recent comprehensive systematic review and meta-analysis indicating that its efficacy is mostly for bigger stones (Wang et al., 2017). However, in recent years, several significant randomized double-blind clinical trials have cast doubt on these recommendations (Pickard et al., 2015; Meltzer et al., 2018; Ye et al., 2018). Nifedipine, a calcium channel blocker, inhibits calcium influx and endogenous prostaglandin production, which lessens the human ureter’s spontaneous rhythmic contractions and may be advantageous for MET (Dellabella et al., 2005). Comparatively, it has limited effect on ureter smooth muscle compared with α-blockers (Wang et al., 2016). Furthermore, as compared to placebo- or tamsulosin-treated individuals, nifedipine was linked with a greater number of side effects such as nausea and vomiting, headache, and sleepiness (Porpiglia et al., 2004). As a result, the utilization of calcium channel blockers is currently uncommon. PDE5 inhibitors, such as sildenafil and tadalafil, are used in the treatment of erectile dysfunction, pulmonary hypertension, and benign prostatic hyperplasia. It is theorized that phosphodiesterase PDEI-5 increases cyclic guanosine monophosphate in ureteral smooth muscle, leading to ureteral relaxation and facilitating stone evacuation (Bai et al., 2017; Montes Cardona and García-Perdomo, 2017). However, the value of this class of drug for treatment of calculus is still in its infancy. Diclofenac and celecoxib are the most commonly used NSAIDs for ureteral colic. Although many clinical trials have shown that NSAIDs, in combination with either α-blockers or calcium channel blockers, are an effective treatment, beneficial effects of this drug class appear to be solely pain relief with no effect on calculus expulsion time (Lv and Tang, 2014; Assimos et al., 2016).

Mirabegron is a β3-adrenergic agonist medication used to treat overactive bladder. Matsumoto et al. confirmed the existence of β3-adrenoceptors (ARs) in ureteric urothelial cells and smooth muscle in the lower, middle, and proximal ureters using immunohistochemical methods (Matsumoto et al., 2013; Shen et al., 2017). Crucial smooth muscle relaxation was achieved by activating three receptors, which facilitated stone ejection and demonstrated the effectiveness of mirabegron as a MET. Constipation, dry mouth, dyspepsia, nausea, and other side effects were equally common in the placebo and mirabegron groups during clinical studies for overactive bladder (Chapple et al., 2010; Khullar et al., 2013). Indeed, β3-AR agonists might be regarded as a promising strategy for treating ureteral stones. So far, there is no meta-analysis on randomized controlled trials (RCTs) assessing mirabegron to treat ureteral stones. Therefore, the purpose of this study was to assess the effectiveness of mirabegron as a MET for ureteral stones in adults.

## 2 Materials and methods

### 2.1 Literature search strategy

The systematic review was conducted in accordance with the PRISMA recommendations for reporting items for systematic reviews and meta-analyses, as well as the criteria for Cochrane reviews (Page et al., 2021). We conducted an unrestricted electronic search of PubMed, Embase, the Cochrane Library, Ovid, Scopus, and trial registries from inception to November 2023. Mirabegron or beta-3 agonist, ureteral calculi or ureterolithiasis or ureteral stones, medical expulsive treatment, and randomized controlled study were the search phrases utilized. To eliminate omissions, studies mentioned in relevant publications were also assessed.

### 2.2 Study selection and exclusion criteria

The studies used for the meta-analysis matched the following criteria (Hollingsworth et al., 2006): randomized controlled trials (RCTs) (Huang et al., 2016); research only included individuals with ureteral stones that were 10 mm or smaller (Liu et al., 2015); all patients had a kidney–ureter–bladder (KUB) X-ray and an abdominal ultrasound for a preliminary diagnosis; if necessary, a computed tomography (CT) scan was performed (Wang et al., 2017); mirabegron was compared to placebo (Ye et al., 2018); and data that are complete and ready for analysis.

The exclusion criteria were the following (Hollingsworth et al., 2006): study participants were not adults (younger than 18 years) (Huang et al., 2016); patients with a urinary tract infection (UTI), numerous or bilateral ureteral stones, radiolucent ureteral stones, a solitary kidney, pregnancy, severe hydronephrosis, or renal insufficiency (Liu et al., 2015); patients with a history of ureteral or endoscopic surgery in the past (Wang et al., 2017); patients in the use of alpha-blockers or calcium channel blockers, or severe hypertension (Ye et al., 2018); studies without available data (Pickard et al., 2015); studies with duplicated data (Meltzer et al., 2018); and studies updated in subsequent publications.

### 2.3 Data abstraction

The literature screening, appraisal, and data extraction were carried out independently by two co-authors, and all issues were addressed and resolved by a third, independent author. The extracted content included study characteristics (first author, publication year, study area, and cases in each group), patient characteristics (age, location and size of the stone, stone sizes, control (placebo), follow-up time, and intervention), method (randomization, allocation concealment, participant and outcome assessment blinding, incomplete outcome data, selective reporting result, and other potential biases), and outcomes (stone expulsion rate, stone expulsion interval, and pain episodes). The quality of all included studies was evaluated. All of the above material was double-checked, and any discrepancies were resolved through conversation.

### 2.4 Bias risk assessment

We employed the Cochrane Collaboration’s risk of bias technique, which encompasses areas such as randomization, allocation, and concealment, blinding of participants and workers, blinding of outcome assessors, inadequate outcome data, reporting bias, and other biases (Higgins et al., 2011). Each of these seven areas was categorized as: “low risk of bias,” “unclear risk of bias,” or “high risk of bias."

### 2.5 Statistical analysis

The statistical analyses were carried out using Review Manager 5.4 and Stata 17. Continuous variables were reported as the mean difference (MD) with a 95% confidence interval (CI), whereas dichotomous variables are provided as odds ratio (OR) with a 95% CI. The I^2^ statistic and the *p*-value tests were used to assess heterogeneity. An I^2^ > 50% was considered heterogeneous, and a sensitivity analysis was undertaken to explore the cause of heterogeneity; if necessary, a random-effects model was used to compare data. A fixed-effects model was utilized for analysis when heterogeneity was regarded minimal (I^2^ < 50%). In addition, an inverted funnel plot was drawn to evaluate the possibility of publication bias. The results were considered statistically significant when *p* < 0.05.

## 3 Results

### 3.1 Study selection

**FIGURE 1:**
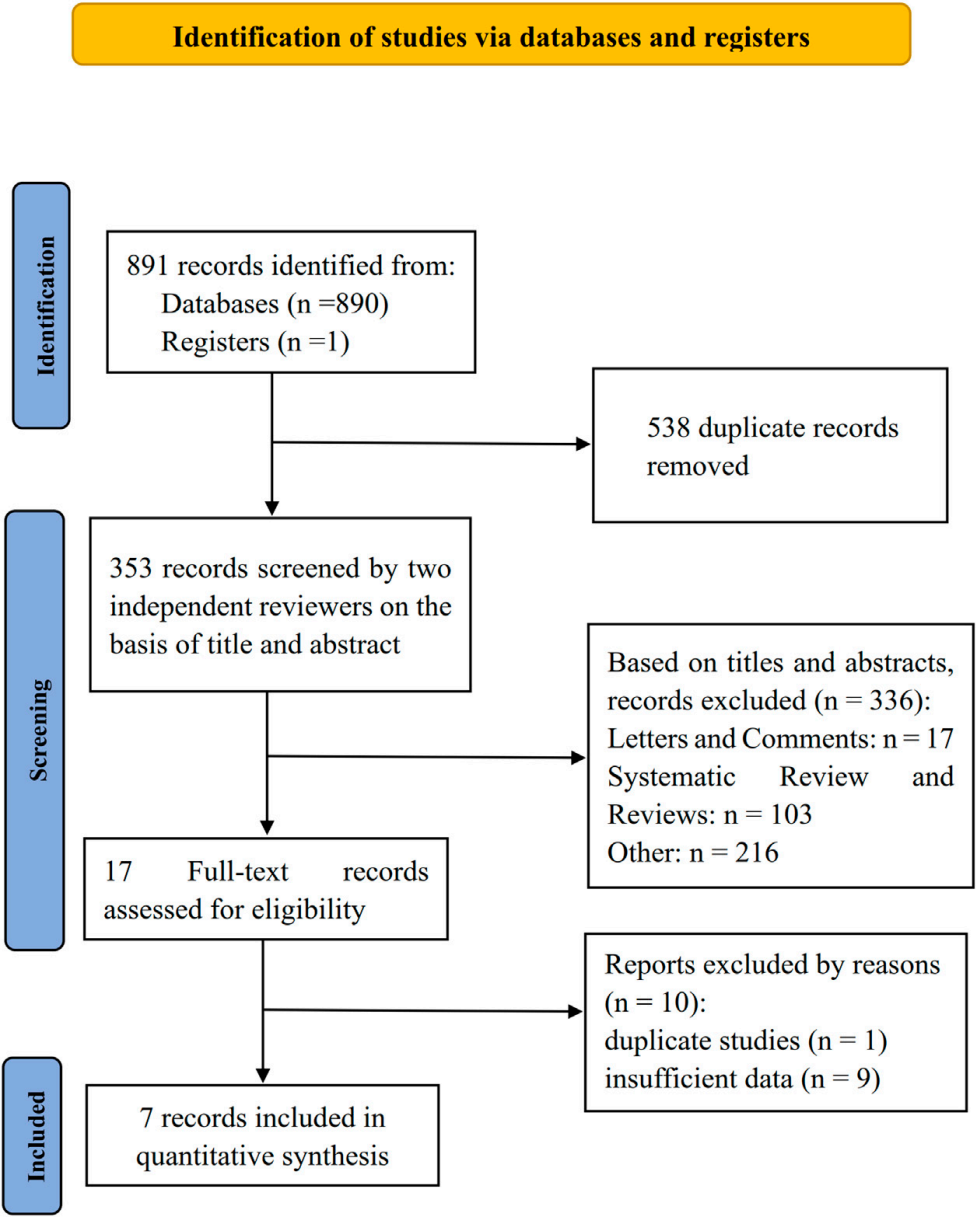
PRISMA of the selection process.

We found 890 records in databases and register searching. Following the elimination of duplicated entries, 352 references were screened for title and abstract, providing 10 possibly relevant references that were further studied. One study was duplicated, and two conference abstracts were excluded due to insufficient available information to draw conclusions (Figure 1).

### 3.2 Study characteristics

**FIGURE 2:**
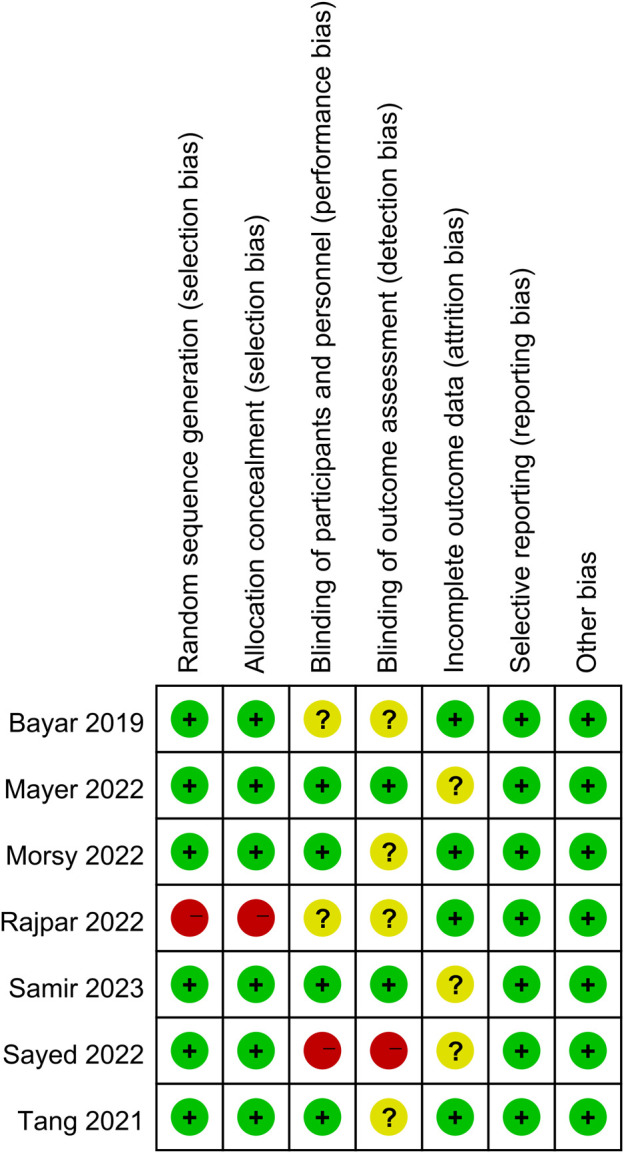
Risk of bias summary. Note: (+) low risk, (?) unclear risk, and (−) high risk.

Study characteristics are summarized in Table 1, comprising 701 participants. All studies were RCTs (Bayar et al., 2020; Abdel-Basir Sayed et al., 2022; Mayer, 2022; Morsy et al., 2022; Rajpar et al., 2022; EAU, 2023; Samir et al., 2023), which had accrued between September 2009 and November 2022 (Figure 1). These studies were conducted in the United States, Pakistan, Turkey, Egypt, China, and Saudi Arabia. The age of the participants ranged from 28.7 to 47.3 years. The average size of the stones varied from 5.4 to 7.3 mm. Except for differences in the baseline age between the control and experimental groups in Morsy et al., sex, age, degree of hydronephrosis, and stone size did not significantly differ between the groups. In terms of stone location, four studies included DUS patients, while three others also included non-DUS cases. Most trials diagnosed ureteral stones using a kidney–ureter–bladder (KUB) X-ray, ultrasound, or CT, and the majority employed a combination of patient history, KUB X-ray, US, and/or CT to assess the stone passage. In all studies, the dropout rate was minimal. The follow-up period was 4 weeks or 30 days. In one trial, the control group received tamsulosin, whereas the experimental group received mirabegron with tamsulosin. In other trials, the control group received a placebo or a pain reliever. The experimental group received a daily dose of 50 mg mirabegron. The key finding of all included research was SER, which was assessed by the lack of a ureteral stone on imaging, either CT or KUB X-ray, at the end of the study period. Stone ejection time (days) and pain episodes were secondary outcomes. Figure 2 depicts the risk of bias.

**TABLE 1:** Specifications of each study included in the meta-analysis.

Study, year	Country	Participant	Experimental group	Control group	Follow-up	Diagnostic method	Sample, N = 701
Bayar et al. (2020)	Turkey	Ureteral stones 4–10 mm	Mirabegron	Placebo	4 weeks	US and/or KUB	115
Morsy et al. (2022)	Egypt	DUSs ≤10 mm	Mirabegron + diclofenac	Diclofenac	30 days	KUB and US or CT	50
Rajpar et al. (2022)	Pakistan	Ureteral stones ≤10 mm	Mirabegron + diclofenac	Diclofenac	4 weeks	KUB and US and CT	200
Tang et al. (2021)	China	DUSs ≤10 mm	Mirabegron + tamsulosin	Tamsulosin	4 weeks	KUB and US and CT	90
Abdel-Basir Sayed et al. (2022)	Saudi Arabia	DUSs ≤10 mm	Mirabegron + ketorolac	Ketorolac	4 weeks	US and KUB and NCCT	96
Mayer (2022)	United States	Ureteral stones ≤10 mm	Mirabegron	Placebo	30 days	CT or US and KUB	33
Samir et al. (2023)	Egypt	DUSs 5–10 mm	Mirabegron	Placebo	4 weeks	KUB and US and NCCT	117

Abbreviations: US, ultrasound; KUB, kidney–ureter–bladder X-ray; DUSs: distal ureteral stones; CT, computed tomography; NCCT, non-contrast computed tomography.

### 3.3 Results of individual studies

#### 3.3.1 Stone expulsion rate (SER)

**FIGURE 3:**
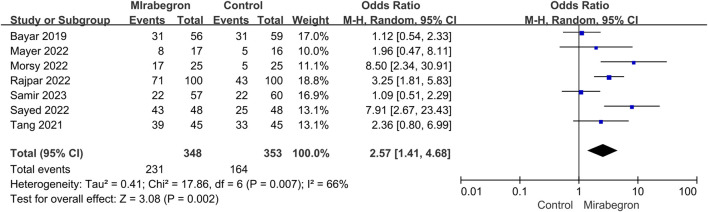
Forest plot: stone expulsion rate (SER) between the mirabegron and the control groups when the ureteral stone was smaller than 10 mm. CI, confidence interval; df, degrees of freedom; M–H, Mantel–Haenszel.

**FIGURE 4:**
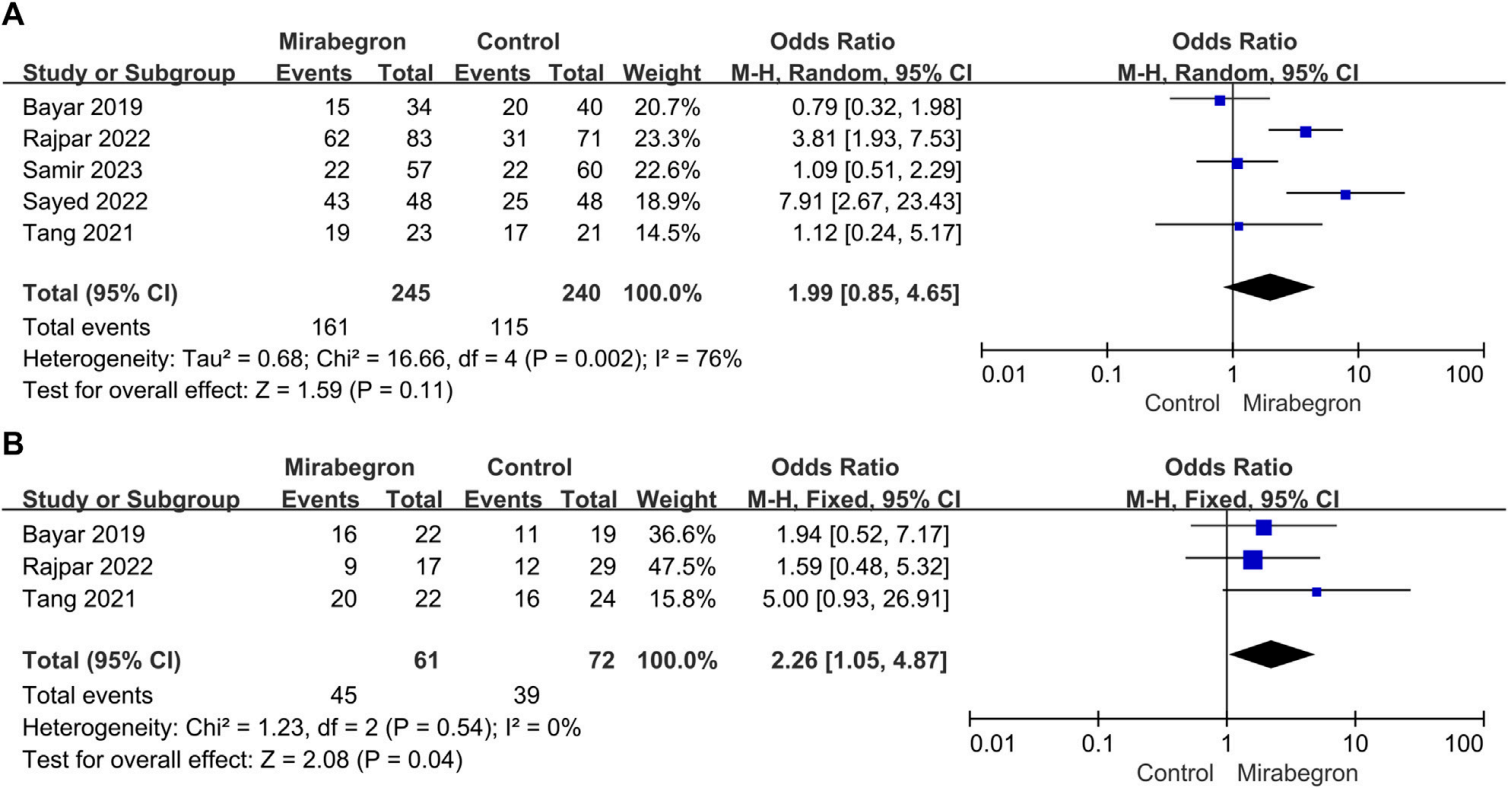
**(A)** Forest plot: SER for patients with large stones (>4–6 mm) between the mirabegron and control groups. CI, confidence interval; df, degrees of freedom; M–H, Mantel–Haenszel. **(B)** Forest plot: SER for patients with small stones (≤4–6 mm) between the mirabegron and control groups. CI, confidence interval; df, degrees of freedom; M–H, Mantel–Haenszel.

**FIGURE 5:**
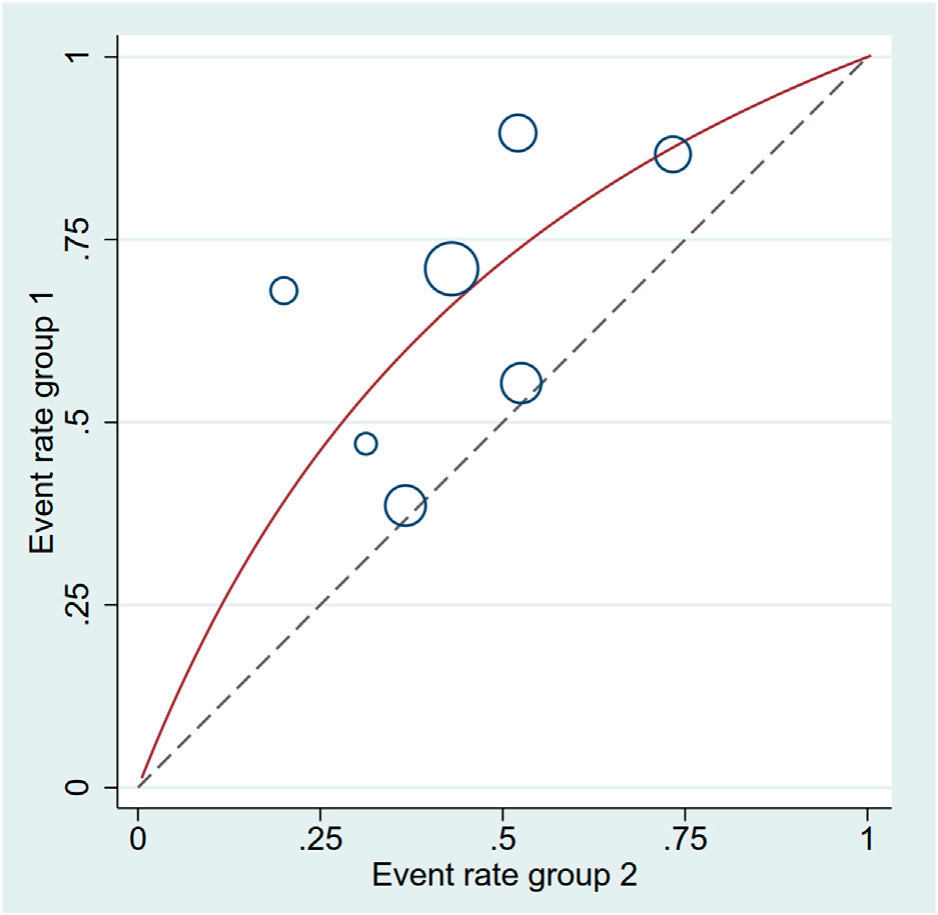
L’Abbe plot: the effect of mirabegron on the SER.

**FIGURE 6:**
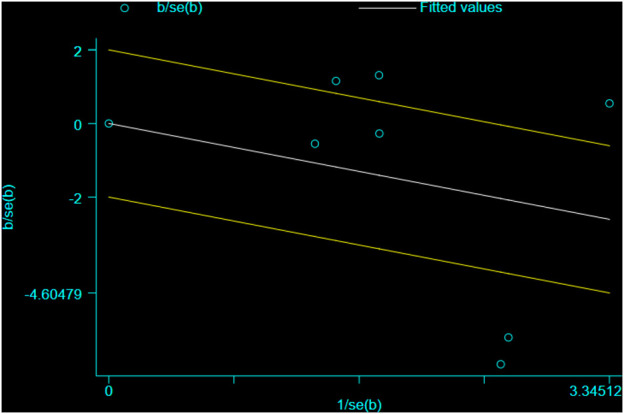
Galbraith plot: the efficacy of mirabegron in the SER using a random-effects model.

**FIGURE 7:**
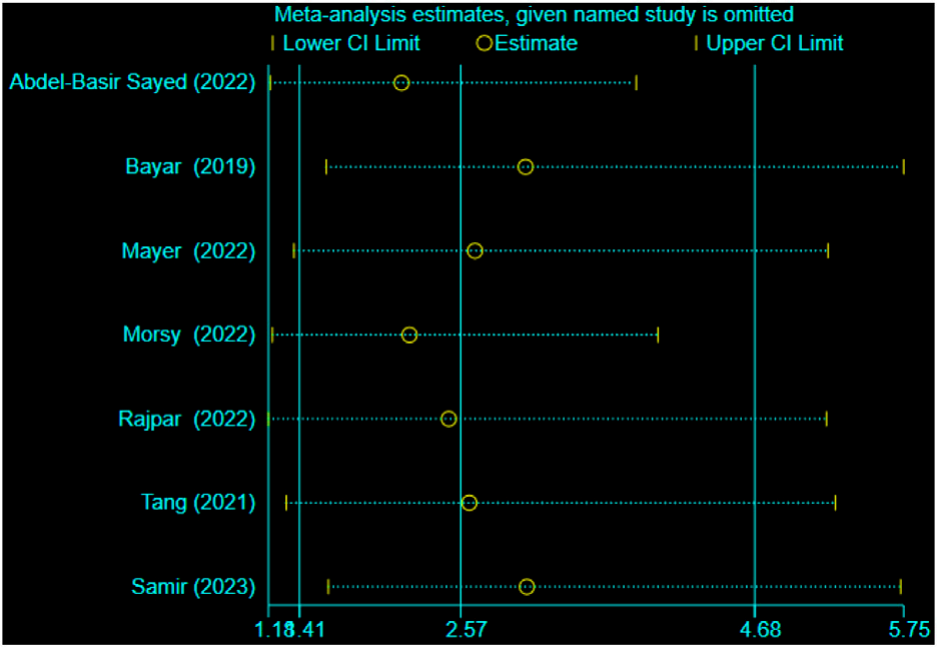
Sensitivity analysis between mirabegron and SER.

In all trials (701 individuals, of which 348 were in the mirabegron group and 353 in the control group), mirabegron-treated patients displayed a higher SER (OR = 2.57, 95% CI = 1.41–4.68, *p* = 0.002) than control ones when the ureteral stone was smaller than 10 mm (Figure 3). There was, however, variability between trials (*p* = 0.007, I^2^ = 66%). Figures 4A, B show a forest plot of all studies sorted by stone size, indicating that changes in stone size can explain some of the variability. In addition, the L’Abbe and Galbraith plots indicate an important heterogeneity in one study (Figures 5, 6). Therefore, the sensitivity analysis of all included literature studies found that the study of Bayar had a great impact on the heterogeneity of the results (Figure 7). In the discussion, we will explore the possible causes of heterogeneity.

#### 3.3.2 Large stones

SER in the subgroup of patients with big stones (minimum stone size of 4–6 mm) was estimated in a subgroup analysis (Figure 4A). Five of the seven studies were included in this subgroup analysis; however, the definition of stone size differed between studies, with some using a cutoff of 5 mm and others using 4 or 6 mm. For patients with large stones, the meta-analysis indicated no discernible difference between the control and the mirabegron groups in a random-effects model (OR = 1.99, 95% CI = 0.85–4.65, *p* = 0.11).

#### 3.3.3 Small stones

Three of the seven trials (Figure 4B) showed results for patients with small stones (a maximum stone size of 4–6 mm). Similarly, the definition of stone size differed between studies since some used a threshold of 5 mm, while others used a threshold of 4 or 6 mm. Mirabegron-treated patients showed a higher SER than control ones (OR = 2.26, 95% CI = 1.05–4.87 *p* = 0.04), and no heterogeneity was detected (*p* = 0.54; I^2^ = 0%).

#### 3.3.4 Distal ureteral stones (DUSs)

**FIGURE 8:**
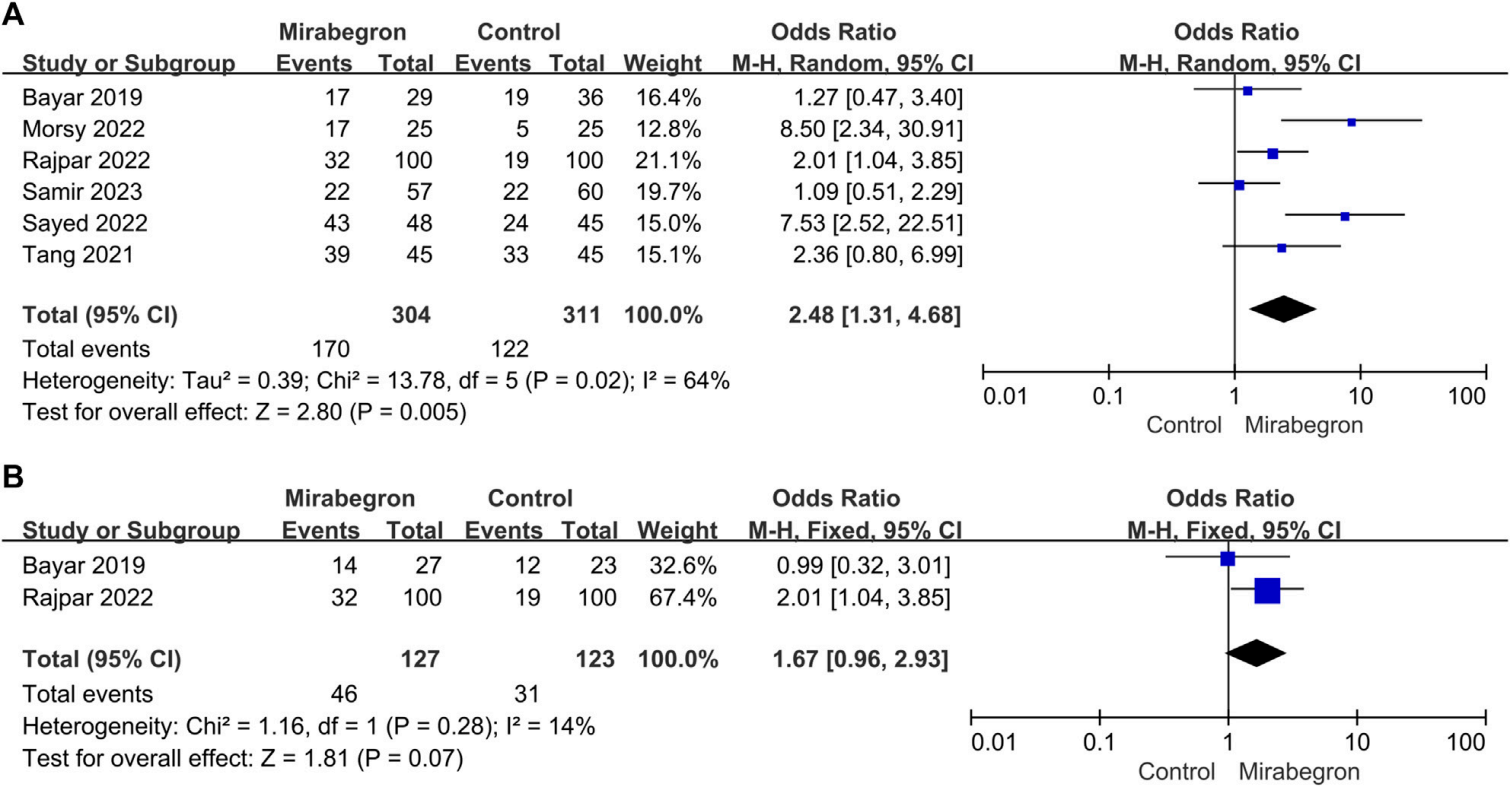
**(A)** Forest plot: SER for patients with distal ureteral stones between the mirabegron and control groups when the ureteral stone was smaller than 10 mm. CI, confidence interval; df, degrees of freedom; M–H, Mantel–Haenszel. **(B)** Forest plot: SER for patients with non-distal ureteral stones between the mirabegron and control groups when the ureteral stone was smaller than 10 mm. CI, confidence interval; df, degrees of freedom; M–H, Mantel–Haenszel.

Six of the seven trials (Figure 8A) reported results for DUSs. These trials were heterogeneous (*p* = 0.02, I^2^ = 64%); thus, a random-effects model was used. It was clear that mirabegron treatment resulted in a higher SER than the control group (OR = 2.48, 95% CI = 1.31–4.68, *p* = 0.005).

#### 3.3.5 Non-distal ureteral stones (non-DUSs)

Two of the seven trials (Figure 8B) reported results for non-DUSs. The meta-analysis using a fixed-effects model showed no discernible difference between the control and the mirabegron groups in terms of SER. (OR = 1.67, 95% CI = 0.96–2.93, *p* = 0.07).

#### 3.3.6 Large vs. small stones in the mirabegron group

**FIGURE 9:**
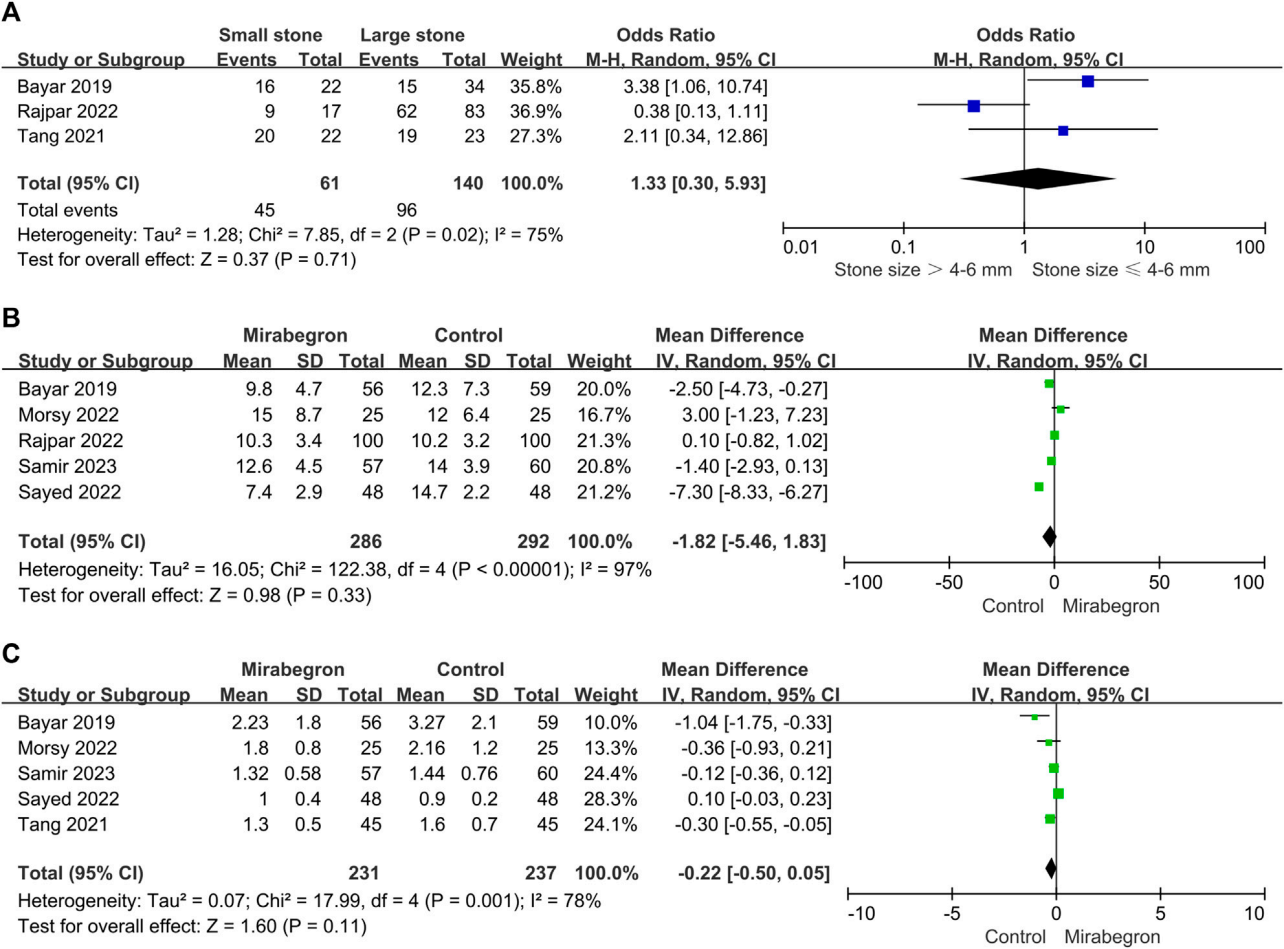
**(A)** Forest plot: SER subgroup analysis in the mirabegron group based on stone size. CI, confidence interval; df, degrees of freedom; SD, standard deviation; IV, inverse variance. **(B)** Forest plot: stone ejection time (days) between the mirabegron and control groups when the ureteral stone was smaller than 10 mm. CI, confidence interval; df, degrees of freedom; SD, standard deviation; IV, inverse variance. **(C)** Forest plot: pain episodes in the mirabegron and control groups when the ureteral stone was smaller than 10 mm. CI, confidence interval; df, degrees of freedom; SD, standard deviation; IV, inverse variance.

In the mirabegron group, three of the seven trials provided results for large stones vs. small stones (Figure 9A). Given the trial heterogeneity (*p* = 0.02, I^2^ = 75%), the random-effects model revealed no significant difference in SER among patients with a large or small stone (OR = 1.33, 95% CI = 0.30–5.93, *p* = 0.71).

#### 3.3.7 Stone expulsion time (days)

Five of the seven trials reported stone expulsion time outcomes (Figure 9B). There was heterogeneity across trials (*p* < 0.00001, I^2^ = 97%), and in a random-effects model, we found no significant difference in stone ejection time between the mirabegron and the control groups (standard mean difference = −1.82, 95% CI = −5.46–1.83, *p* = 0.33).

#### 3.3.8 Pain episodes

Five of the seven trials reported pain episode outcomes (Figure 9C). There was heterogeneity across trials (*p* = 0.001, I^2^ = 78%); thus, a random-effects model was used. There were no obvious differences in pain episodes between groups (standard mean difference = −0.22, 95% CI = −0.50–0.05, *p* = 0.11).

### 3.4 Risk of bias in studies

**FIGURE 10:**
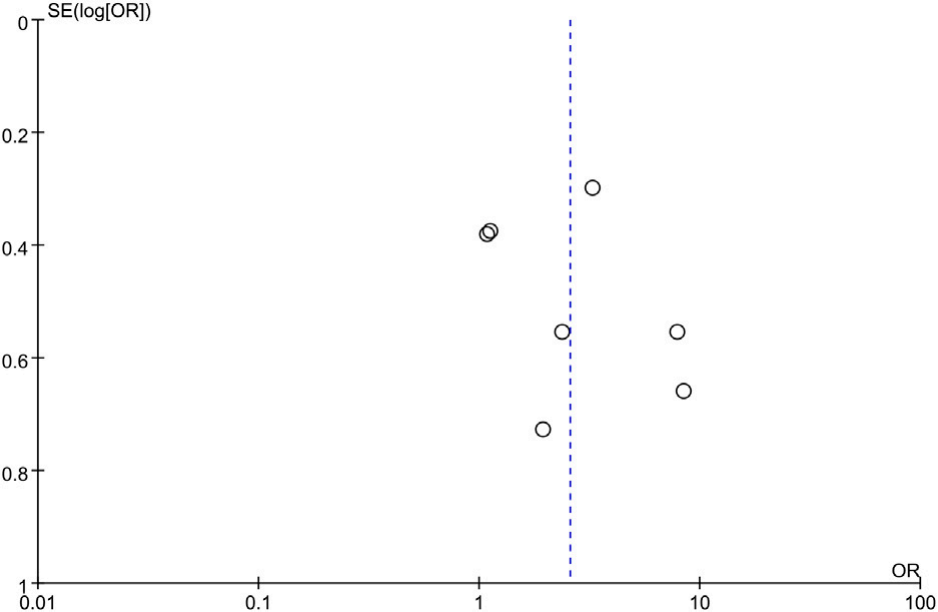
An inverted funnel diagram was drawn to investigate the publication bias.

The geometries of inverted funnel plots revealed a minimal likelihood of publication bias in the SER results between the mirabegron and the control groups when the ureteral stone was smaller than 10 mm (Figure 10).

## 4 Discussion

According to the most recent EAU guidelines, MET appears to be effective for treating patients with ureteral stones who are receptive to conservative care (EAU, 2023). The efficacy of the most widely used α-adrenoceptor blocker in MET has been confirmed by multiple meta-analyses (Yilmaz et al., 2005; Wang et al., 2017; Hsu et al., 2018; Liu et al., 2018). However, there are conflicting data between these trials, and many well-designed, multicentric, placebo-controlled, double-blind randomized studies demonstrate little or no effect, with the exception of a moderate benefit for DUSs >5 mm (Pickard et al., 2015; Sur et al., 2015; Furyk et al., 2016; Ye et al., 2018). Furthermore, patients utilizing alpha-adrenergic blockers may experience adverse effects, including dry mouth, dizziness, nausea, palpitation, retrograde ejaculation, and orthostatic hypotension, owing to the inherent pharmacological mechanisms involved (De Coninck et al., 2019).

Mirabegron is currently mostly used to treat overactive bladder, and reviews and meta-analyses indicate that the therapeutic dose of mirabegron, 50 mg, is not associated with blood pressure changes, QT time, or heart rate (Michel and Gravas, 2016; Geoffrion, 2017; Deeks, 2018). Smooth muscle cells make up the majority of the muscular tube that makes up the mature human ureter. Urine is propelled from the kidneys to the bladder by waves of smooth muscle contraction and relaxation traveling down the muscular tube, which is the main purpose of this organ (Woolf and Davies, 2013). The relaxant effect of β3 agonists in the ureter has been shown in several *in vivo* studies in dogs and pigs, where the intraluminal pressure in the ureteral has significantly decreased (Wanajo et al., 2011). Mirabegron appears to be effective in the treatment of ureteral stones, according to a number of RCTs conducted in recent years. Seven RCTs with a total of 701 patients were combined for this meta-analysis. After 4 weeks, mirabegron treatment substantially enhanced the SER (OR = 2.57) of ureteral stones up to 10 mm, as compared to the control group. SER was 67% and 46% in the mirabegron and the control groups, respectively. However, there was no statistically significant change in stone ejection time or discomfort events during the follow-up period. Clinically, the location and size of ureteral stones are important prognostic factors concerned by doctors. Therefore, we performed a subgroup analysis to assess these factors. We found that mirabegron can significantly improve the SER (OR = 2.26) of patients with small stones. In terms of DUSs, mirabegron-treated patients achieved a higher SER than the control group. However, for non-DUSs, no significant differences were observed between the groups. There was also no discernible difference in the SER of mirabegron-treated patients stratified according to their stone size.

There is little evidence on spontaneous ureteral stone transit based on the stone size (Skolarikos et al., 2010). Moreover, 95% of ureteral stones smaller than 4 mm are expected to be expelled within 40 days (Preminger et al., 2007). Based on present data, a definitive cut-off size for stones that are likely to pass spontaneously cannot be determined (Preminger et al., 2007). The definition of stone size in the papers herein included varied since some studies used a threshold of 5 mm, while others used 4 or 6 mm. Therefore, stones smaller than 4–6 mm are defined as small stones; otherwise, they are defined as large stones. In all cases of ureteral stones <10 mm, mirabegron treatment clearly improved SER (OR = 2.57, 95% CI = 1.41–4.68). However, there was heterogeneity across the studies (*p* = 0.007, I^2^ = 66%). A subgroup analysis detected stone size as the probable cause of heterogeneity. Mirabegron treatment led to a higher SER in patients with small ureteral stones (OR = 2.26, 95% CI = 1.05–4.87), and no heterogeneity was seen across these trials (*p* = 0.54; I^2^ = 0%). Mirabegron improved SER by 20% (74% vs. 54%) compared to the control group. Nevertheless, mirabegron did not show any notable benefits in patients with a large stone. In addition, based on the sensitivity analysis of included studies, it was found that the study of Bayar had a great influence on the heterogeneity of our meta-analysis. SER is affected not only by the size and location of stones but also by the degree of ureter spasm or edema and the degree of hydronephrosis. Shen et al. observed that ureter dilatation lowered the expression of all β-AR subtypes in human ureter mucosa and muscle layers. In the early stages of the illness, the use of highly selective β3-AR agonists may improve symptoms of ureteral smooth muscle spasm. However, in latter stages, when compensation in the ureter lesions has been lost, β3-AR agonists are no longer appropriate therapy strategies (Shen et al., 2017). Bayar et al. did not compare differences in the time of illness and the degree of hydronephrosis, which could partially explain the heterogeneity we detected. A previous meta-analysis conducted by Cai et al. (2022) found that mirabegron significantly improved the SER of small stones, while no statistical difference was found for large stones (Cai et al., 2022). Although the results of our meta-analysis are the same as those of Cai et al., our study is more reliable because all included evidence comprised RCTs. As for stone expulsion time, mirabegron treatment was not beneficial.

The stone passage was spontaneously reported in 49% of upper ureteral stones, 58% of mid ureteral stones, and 68% of distal ureteral stones (Yallappa et al., 2018). In our study, six of the seven studies reported the results of SER for DUSs. When compared to the control group, mirabegron dramatically improved SER of these patients (56% vs. 39%). In terms of pain episodes, we did not detect benefits of mirabegron treatment, contrary to the findings of Cai et al. (2022).

Interestingly, in the study by Morsy et al., the patients were divided into the following three groups: 30 patients received mirabegron 50 mg + diclofenac Na 100 mg tab daily, 30 patients received tamsulosin HCL 0.4 mg cap + diclofenac Na 100 mg tab, and 30 patients received diclofenac Na 100 mg tab alone. Diclofenac Na was administered to all groups just for pain relief. For ureteral stones <10 mm, 68% of patients in group A expelled the stone during therapy, compared to 60% and 20% in groups B and C, respectively (Morsy et al., 2022). Furthermore, based on the data on Tang et al., in patients with 5-mm stones, the experimental group (mirabegron 50 mg once daily plus tamsulosin 0.2 mg once daily) had a higher SER than the control group (tamsulosin 0.2 mg once daily) (Abdel-Basir Sayed et al., 2022). Although mirabegron appears to demonstrate a similar or even superior stone expulsion effect compared to tamsulosin, further large-scale RCTs are essential to substantiate these findings.

According to Song et al. (2016), the coronal length (craniocaudal) and axial stone diameter were both significantly predictive of the degree of hydronephrosis (ANOVA, *p* < 0.001 for both) (Song et al., 2016). The average axial diameter of the stones classified by the degree of hydronephrosis was 3.0 mm for no hydronephrosis, 3.9 mm for light hydronephrosis, 4.9 mm for moderate hydronephrosis, and 12.7 mm for severe hydronephrosis. Mirabegron may be more effective in facilitating the extrusion of tiny stones because of the dilated ureter’s reduced expression of β-AR. Additionally, 176 individuals with a single obstructive ureteral stone (111 distal ureteral calculi and 65 proximal ureteral calculi) were examined by Eisner et al. The axial calculus diameter of the proximal and distal stones did not differ from one another (mean 5.3 mm vs. 5.0 mm, respectively, *p* = 0.29), and the proximal ureteral stones were linked to a higher degree of ureteral dilatation than the distal stones (mean 6.1 mm vs. 5.3 mm, respectively, *p* = 0.01) (Eisner et al., 2008). The aforementioned results might help explain the mirabegron-induced notable rise in the incidence of small stones and distal ureteral stones.

A single dose of 50 mg mirabegron per day is safe. Mirabegron had fewer adverse drug reactions in all studies. There were only two cases of hypertension, two headaches, one orthostatic event, two nasal blockages, and five occurrences of dizziness recorded.

The main limitations to this study are as follows (Hollingsworth et al., 2006): the quality of evidence supporting the use of mirabegron for MET raises some concerns. Some studies failed to blind the participants or the outcome assessment (Huang et al., 2016). The small sample size of some studies may affect the accuracy of the results (Liu et al., 2015). There are differences in inclusion criteria, exclusion criteria, research methods, and statistical methods among different studies, which may lead to high heterogeneity (Wang et al., 2017). The inability to evaluate the ureteral wall thickness (UWT), ureteral diameter (UD), and the ratio of ureter-to-stone diameter (USD) hampered the comparison of SER (Samir et al., 2021; Selvi et al., 2021; Ye et al., 2018). Although several factors, such as the degree of hydronephrosis, the time of illness, MET compliance, exercise volume, and various follow-up durations, might influence SER, they were not included in our subgroup analysis (Pickard et al., 2015). The use of non-steroidal anti-inflammatory medicines may have an effect on the outcomes since they can alleviate ureteral edema (Holdgate and Pollock, 2004).

In summary, the current meta-analysis found that mirabegron was superior to placebo in terms of efficacy for the treatment of ureteral stones, particularly stones ≤4–6 mm and DUSs. High-quality multicenter RCTs are needed to corroborate these findings. More realistic findings could be obtained if the researchers compare the effectiveness of mirabegron in removing stones based on the degree of hydronephrosis and the time of illness.

## Data Availability

The original contributions presented in the study are included in the article/Supplementary Material; further inquiries can be directed to the corresponding author.
